# A Smart Collaborative Routing Protocol for Reliable Data Diffusion in IoT Scenarios

**DOI:** 10.3390/s18061926

**Published:** 2018-06-13

**Authors:** Zheng-Yang Ai, Yu-Tong Zhou, Fei Song

**Affiliations:** 1 School of Electronic and Information Engineering, Beijing Jiaotong University, Beijing 100044, China; 17111017@bjtu.edu.cn; 2 Institute of Education and Economy Research, University of International Business and Economics, Beijing 100029, China; zhouyutong@uibe.edu.cn

**Keywords:** geographic routing protocol, inspecting node mechanism, reliable data diffusion, game theory, IoT

## Abstract

It is knotty for current routing protocols to meet the needs of reliable data diffusion during the Internet of Things (IoT) deployments. Due to the random placement, limited resources and unattended features of existing sensor nodes, the wireless transmissions are easily exposed to unauthorized users, which becomes a vulnerable area for various malicious attacks, such as wormhole and Sybil attacks. However, the scheme based on geographic location is a suitable candidate to defend against them. This paper is inspired to propose a smart collaborative routing protocol, Geographic energy aware routing and Inspecting Node (GIN), for guaranteeing the reliability of data exchanging. The proposed protocol integrates the directed diffusion routing, Greedy Perimeter Stateless Routing (GPSR), and the inspecting node mechanism. We first discuss current wireless routing protocols from three diverse perspectives (improving transmission rate, shortening transmission range and reducing transmission consumption). Then, the details of GIN, including the model establishment and implementation processes, are presented by means of the theoretical analysis. Through leveraging the game theory, the inspecting node is elected to monitor the network behaviors. Thirdly, we evaluate the network performances, in terms of transmission delay, packet loss ratio, and throughput, between GIN and three traditional schemes (i.e., Flooding, GPSR, and GEAR). The simulation results illustrate that the proposed protocol is able to outperform the others.

## 1. Introduction

The rapid evolution of Internet of Things (IoT) promotes the life quality and work efficiency for human [1]. The IoT is a creative pattern of connecting various things, which contains two meanings. First, the core and foundation of the IoT are still the network, which is an extension and expansion of the Internet. Second, the end users are extended to any items for information exchange and communication, i.e., the connection of objects. Due to the sensing function and wireless communication capability, the applications of IoT are providing broad prospects. As a pervasive technology, it has been widely used in vehicle monitoring, health care, urban transportation, space exploration and other promising fields [2]. As the most representative member of IoT, the Wireless Sensor Network (WSN) can serve as a general platform for many domains, such as measurement of various environmental parameters (temperature, pressure, humidity, light, etc.). For the commercial and military applications, secure and reliable communications are crucial for maintaining data dependability and confidentiality [3]. In recent years, many security aspects in WSN have been reconsidered, such as routing, localization, data fusion and access control [4,5,6,7]. We take the secure routing as an example. As the foundation of the information perception and transmission in IoT, the selection of routing strategies is an important part for the security defense and efficient delivery [8,9,10,11]. The design process of a routing protocol needs to make tradeoff in complexity, energy consumption and other aspects. An excellent routing protocol should resist the illegal intrusions and malicious destructions without affecting the normal packet exchanging.

As an eligible member of security routing protocols, the geographic routing protocols forward the request packets to a specified location by utilizing the location information of nodes, which shortens the scope of data transmission. Two typical schemes are Greedy Perimeter Stateless Routing (GPSR) and Geographic and Energy Aware Routing (GEAR). For the geographic routing protocols, each node can perceive and exchange its own position with the neighbor nodes. Therefore, the forwarding path will be established by referring to the location information.

However, the routing protocols in WSN frequently suffer various attacks, including selective forwarding attack, black hole attack, Sybil attack, wormhole attack, hello flooding attack, ACK attack and so on [12,13]. Among them, the black hole and wormhole attacks are more intractable to be monitored. Although it is effortless for the traditional geographical routing protocols to cope with both attacks, the shared location information of nodes might be intentionally misrepresented or maliciously modified by Sybil attacks. Therefore, a reliable, stable and effective routing protocol is urgently needed in the current WSN.

Motivated by above considerations, we aim to focus on the routing and transmission scheme to optimize network performances. First, a novel geographical routing protocol, Geographic energy aware routing and Inspecting Node (GIN), is proposed, which integrates the GEAR protocol with the inspecting mechanism. On the one hand, the GEAR protocol can easily establish the routing path by leveraging the local cooperation information of nodes, rather than requesting them from the base station. According to the geographical information of nodes, the location of so-called “neighbors” can be found, which might be further than the spread range of normal signals. Thus, it is quite hard for an attacker to form a sewage pool. Once a wormhole attack is launched to affect traffic, the behaviors will be immediately dectected. On the other hand, the new idea of combining with Inspecting Node (IN) can deploy the reputation scheme to restrain the Sybil and selfishness attacks [14]. The inspecting nodes can be selected through the game theory. Finally, the network model is established and the specific simulation results are provided. We demonstrate that the proposed protocol is able to optimize delay, packet loss ratio and throughput. Furthermore, the system security is improved.

The main contributions of this paper could be summarized in three aspects:

(1) A novel geographical routing protocol GIN is proposed by combining the GEAR with the inspecting mechanism. It can resist the typical malicious attacks (black hole, wormhole and Sybil attacks) and promote the system performances.
(2) We make use of the Subgame Perfect Nash Equilibrium (SPNE) to choose the inspecting nodes. In order to maximize the network lifespan, the analysis is based on a non-cooperative and repeated general-sum game to make the tradeoff between costs and capabilities.
(3) The network model and specific routing process are introduced based on the theory analysis. The validation results of NS3 simulator demonstrate that the proposed protocol outperforms the other three protocols in many aspects.

The rest of paper is organized as follows: In Section 2, the related work is presented and discussed from three different perspectives. In Section 3, the specific details of GIN are introduced. Three main aspects include the preliminary foundation, model establishment and implementation processes. In Section 4, the simulation scenario based on NS3 and Wireshark tools is given to explore the advantages of GIN. The validation results are analyzed in depth. In Section 5, we summarize the whole paper and describe the future work.

## 2. Related Work

As the routing protocol plays an increasingly important role in communication areas, more scholars are attracted to investigate the issues in IoT scenarios. In this part, some existing work related with the data transmission is presented. Generally, they can be divided into three categories.

### 2.1. Routing Protocols Based on Geographic Location

For the routing protocols based on geographic locations, the routing path is selected by referring to the nodes’ location information instead of the routing tables, which shortens the transmission range of nodes and extends the network life-cycle. Karim and Nasser [15] raised a Location-aware and Fault tolerant Clustering Protocol for Mobile WSN (LFCP-MWSN) to optimize energy consumption and reduce the end-to-end transmission delay. A fault tolerant mechanism was combined with the routing protocols to identify failures of data links and sensor nodes. However, the proposed protocol was not particularly better than other protocols in terms of end-to-end delay. The simulation results of them were almost identical. Moreover, the Cluster Head (CH) were considered ideally that they would not move out of the cluster in the current round. Srivastava and Sudarshan [16] focused on the efficient management of sensing energy and computational capabilities of network. A novel energy-efficient algorithm ZEEP (Zone based on Energy Efficient routing Protocol) was proposed to improve the packet delivery ratio and enlarge the network life-cycle. The proposed protocol can be applied to both stationary and mobile nodes, without super abundant computation and mechanisms. However, the issues of network security, which could subject to malicious attacks, was not considered by the authors. In [17], Nadeem et al. presented a gateway-based and energy-efficient routing protocol, i.e., M-GEAR for optimizing the energy consumption and network life-cycle. First, the sensor nodes were divided into four logical regions on the basis of their locations. The Base Station (BS) was installed out of the sensing area, while the gateway nodes were installed at the center of the sensing area. Finally, the communication mode of nodes was selected based on whether the distance of two equal regions was beyond the threshold distance. The proposed protocol was a multi-hop routing protocol, which improved three performance metrics (i.e., network life-cycle, residual energy and throughput). A new geographic routing protocol was presented in [18] by levering the energy-sensing technology. The authors focused on the Quality of Service (QoS) to analyze the n-hop neighborhood information, which satisfied the demand of network in real-time video surveillance. The final results showed that the proposed protocol was able to make a better decision than the greedy forwarding. However, more parameters should be taken into account in the simulation. Inspired by GAF (Geographic Adaptive Fidelity), Kaur and Gujral [19] modified the selection criteria of the active node sequence in the discovery phase of an optimized GAF. The selection of the active nodes was based on higher residual energy and less distance from BS. After the network was divided into multiple grids, the chosen active node of each grad can sense data. The simulation results demonstrated that the proposed protocol improved energy efficiency and extended the network life-cycle. In [20], a modified geographical energy aware routing was presented in WSN. The gateway node and the approaches for CH selection were integrated together to optimize energy consumption efficiency and enlarge the network life-cycle. The CH selection was based on the probability and residual energy of nodes. The network was divided into four parts, where the two regions made use of direct communication and the rest of regions adopted the clustering hierarchy. Finally, the sensor nodes in WSN were better distributed. Li et al. [21] presented an Energy-Balanced Greedy forwarding Routing (EBGR) protocol based on the geographical routing protocols to balance the energy consumption of routing paths. The routing path was established by combing the residual energy levels of nodes with the remaining hop counts, instead of considering the Euclidean distance, which optimized the energy efficiency and improved the network life-cycle. Although the EBGR protocol showed longer network latency, it was still acceptable in the wide-area agricultural environments. The simulation results illustrated that the proposed protocol had less computational complexity. Unlike the terrestrial WSN, radio waves cannot be applied in the communications of Underwater Wireless Sensor Network (UWSN). However, the acoustic channels can be used for it. In [22], three main location-based routing protocols (i.e., VBF, HH-VBF and FBR) in UWSN were compared on the basis of data delivery ratio, the quantity of live nodes, end-to-end delay and total energy consumption. For the end-to-end delay and data delivery ratio, the HH-VBF was superior to other protocols. In terms of the alive nodes quantity and total energy consumption, the VBF was outperforming the other two protocols. Sun et al. [23] introduced a novel position-based routing protocol named Speed Up-Greedy Perimeter Stateless Routing (SU-GPSR) protocol in WSN. The main idea was to combine the speed-up mode with the greedy mode to provide a solution in data transmission. Moreover, the proposed next hop selection could be applied to both still and mobile nodes considering energy consumption. Finally, the end-to-end delay, hop counts and packet delivery rate were optimized respectively.

### 2.2. Routing Protocols Based on Hierarchy

For the routing protocols based on hierarchy, the network is divided into multiple clusters. Each cluster owns a CH node to control the communication of nodes. The data is transmitted to the gateway node through the aggregated convergence, which reduces the communication traffic and saves network energy. Yadav and Saxena [24] proposed an improved clustering algorithm based on the fuzzy logic, which maximized the life-cycle of the network. An effective CH node selection can drastically reduce energy consumption and extend the network life-cycle. Therefore, the proposed algorithm separated the network into clusters on the basis of energy level, distant from CH and crowdedness. If the scale of the cluster was greater than the threshold value, the cluster was divided into sub-clusters. Then the CH was selected based on the fuzzy logic method. In [25], a novel WSN framework was presented to process the collected sensory data. The authors made use of the Low Energy Adaptive Clustering Hierarchy (LEACH) protocol to improve the performances of the system (i.e., reducing packets quantity and transmission delay). The traffic monitoring, prediction, filtering, compression and decompression were incorporated in the proposed framework. Moreover, the technologies of encryption and decryption were applied in WSN. Fawzy et al. [26] proposed a four-level clustering hierarchy protocol FL-LEACH based on the three-level protocol (TL-LEACH). A Location based Topology Control (LTC) was used to manage the operation of the active nodes in the sensing field, which minimized the intra term of the expended energy. The proposed protocol had the advantages in reducing required energy of the topology and balancing the energy of nodes. In [27], the authors comprehensively discussed and compared the state-of-the-art successors of LEACH protocol. More than 60 related protocols were analyzed, then divided into single-hop and multi-hop communications. The performances of them were compared based on nine different parameters (energy efficiency, overhead, scalability complexity, and so on). Furthermore, the strong and weak points of each protocol were introduced in detail. Alnawafa and Marghescu [28] presented an improved multi-hop technique based on LEACH protocol, i.e., IMHT-LEACH protocol. The traditional MHT-LEACH protocol distributed all the CHs into two levels, while the IMHT-LEACH protocol divided the CHs into more levels. The simulation results revealed that the proposed protocol obviated the drawbacks of the MHT-LEACH, prolonged the network life-cycle, improved the stability and increased the throughput in WSN. Birajdar and Solapure [29] systematically discussed the working principle of the LEACH protocol by using Omnet++ simulator. The authors first introduced some related transmission protocols, then the working principle of LEACH protocol (i.e., set-up phase and steady-state phase) was analyzed in detail. This protocol was tested in message flow and energy consumption through Omnet++. The results illustrated that the LEACH protocol optimized the energy consumption. In [30], the authors focused on the hierarchic routing protocols, i.e., CTP (Collect Tree Protocol) and LEACH. Both protocols were generally applied in the SHM (Structural Health Monitoring) system to detect the damages of the civil structures. The performances of CTP and LEACH protocols were analyzed based on the cooja simulator. The results showed that the distance of nodes in both CTP and LEACH could affect the performances of SHM systems. Rishikesh et al. [31] analyzed recent researches in LEACH protocol and various improved routing protocols comprehensively, and had the advantages and disadvantages of them discussed in detail. The evaluation of various LEACH protocols was given in the forms of clustering, aggregation of data, type of mobility and type of scalability. In [32], a novel routing protocol was presented in WSN by combing the LEACH protocol and a heuristic method, which lowered the energy consumption. The proposed protocol LEACH-KANG consisted of the Kangaroo Method-based adaptive routing protocol and LEACH protocol, which utilized the optimal path from CHs to BS to transmit data. The proposed protocol extended the network life-cycle and saved energy. The paper [33] analyzed the LEACH-based protocols and compared their performances in detail. The comparison results were involved in the network life-cycle, energy consumption, CH selection and network type. Finally, the authors summarized some characteristics of LEACH-based protocols. Abushiba et al. [34] proposed a novel LEACH protocol, i.e., CH-LEACH (Clustering Hierarchy LEACH) to reduce energy consumption. The architecture and principle of the protocol were introduced. Then, the simulation results were given based on energy efficiency and network life-cycle. The authors in [35] focused on security issues of LEACH protocols to study attack-defense techniques. After the related LEACH protocols were discussed, some possible attacks were analyzed, such as the hello flood attack, Sybil attack and selective forwarding attack. Finally, the pros and cons of these protocols were listed.

### 2.3. Routing Protocols Based on Data

For the routing protocols based on data, the data fusion issues are taken into account. The cooperation of nodes is used for improving transmission efficiency and saving network energy. Zabin et al. [36] presented a novel energy-aware routing protocol named Reliable and Energy Efficient Protocol (REEP) to form a more reliable and energy-efficient path. The REEP protocol consisted of five elements, i.e., sense event, information event, request event, energy event, threshold value and request priority queue. The authors introduced the advantages of REEP in detail, which included sensing event propagation, information event propagation and request event propagation. The paper [37] introduced a modified SPIN (Sensor Protocol for Information via Negotiation) protocol, i.e., M-SPIN protocol to achieve the energy-saving data dissemination. A new phase named “distance discovery” was added into the SPIN protocol to identify the distances from nodes to the sink nodes. The results revealed that the proposed protocol lowered the energy consumption and prolonged the network life-cycle. El-Bendary et al. [38] proposed a new secure routing protocol based on the recognizable direct diffusion algorithm, which could resist the black hole and acknowledgement-spoofing attacks. The µTESLA (micro Timed, Efficient, Streaming, and Loss tolerant Authentication) algorithm was used for authenticating the acknowledgement messages from the sink nodes to the source nodes. Finally, the proposed protocol achieved better event-delivery and event-dropping ratio. Ahmed and Gregory [39] presented a Sector Based Clustering Routing (SBCR) protocol for the distributed Data-Centric Storage (DCS). Based on the network-wide cluster flow distribution, each sector was assigned a non-overlapping TDMA slot. Moreover, an optimally synchronized routing algorithm was also introduced to reduce updating and querying traffic, end-to-end delay and collisions. Then a cross-layer optimization model was established in the medium access control layer. In [40], the authors studied three routing protocols in WSN, i.e., location-based routing protocols, data-centric routing protocols and hierarchical routing protocols. For the data-centric protocols, the paper introduced the SPIN and Direct Diffusion protocols. The comparison results were listed based on various parameters (scalability, mobility, power required, energy efficiency, and so on). A reliable SPIN protocol was presented by utilizing the selective forwarding to increase the data delivery radio in [41]. When some packets were unacknowledged by the source node, the source node would forward them again. The results showed that the proposed protocol optimized the Packet Delivery Ratio (PDR), throughput and routing overhead. Dutta et al. [42] proposed a modified SPIN protocol to save energy and prolong the network life-cycle. An optimal secure transmission path was selected by efficiently implementing this protocol. The general processes of the proposed protocol included data negotiation, setting up route and transmitting data to nodes. Finally, the routing overhead was reduced and data delivery was guaranteed. Jain and Khan [43] comprehensively analyzed the two prominent data-centric protocols, i.e., DDIFF and SPIN protocols. By using NS2 simulator, NAM (Network Animator), TCL (Tool Command Language) and AWK (post processing script) respectively, the performances of them were given based on the end-to-end delay, throughput, control overhead and PDR. Finally, the DDIFF protocol performed better than the SPIN protocol under the static and mobile environments. Chauhan et al. [44] presented an energy-efficient SPIN protocol based on the Hybrid Multi Hop Clustering Scheme (SHMCS), which combined EEHMCS and SPIN protocols. Each node of EEHMCS could sense data from the surroundings and send it to its corresponding CH node. By building an energy consumption model, the total energy loss could be calculated. In [45], the authors investigated the Direct Diffusion Routing Algorithm (DDRA) in detail. This routing algorithm was based on the direct diffusion and the clustering selection scheme to analyze the network topology and energy level of nodes. Finally, the analysis results demonstrated that the DDRA could improve the network delay and delivery rate in a fixed-size scenario. Ashish et al. [46] presented a review on energy-efficient data centric routing protocols for WSN. The related issues of the protocols were discussed, i.e., fault tolerance, scalability, production cost, operating environment, and so on. Finally, the authors gave the comparison results of six data-centric routing protocols in mobility, power usage, scalability and multi-path aspects.

## 3. The Smart Collaborative Routing Protocol for IoT

The sinkhole attack usually deceives the surrounding nodes to forward the packets to the attacker by releasing the attractive routing information (such as the sufficient energy or the nearest distance). Similarly, the wormhole attack forms a tunnel through a short delay link. Based on this tunnel, the received information is transmitted and replayed between different parts of the network. However, the routing protocols based on geographic location can solve these obstacles well. Since the abnormal transmitting distances far exceed normal signal range, the attacking node will be soon discovered. Moreover, the IN mechanism can monitor the behaviors of CHs and MNs (Member Nodes) in real time and prevent the Sybil attacks. In this section, we first introduce the preliminary foundation of the proposed protocol. Then, specific steps of establishing the model are given based on a widely approved protocol. To further certify the overall performances, the six primary processes are also discussed.

### 3.1. Preliminary Foundation

The proposed GIN protocol is based on the GEAR protocol and IN mechanism. To further support the performances of the GIN protocol, we mainly introduce the working principles of the GEAR protocol and IN mechanism respectively.

#### 3.1.1. GEAR Protocol

The GEAR protocol combines the ideas of the directed diffusion routing and GPSR routing. Moreover, it takes the node energy factor into consideration for solving the uneven energy consumption problem. Each node in GEAR protocol can perceive its own location information and residual energy information, which can be exchanged between all neighbor nodes through a simple Hello message. Finally, an optimal path from the sink node to the event area is established based on these information, which avoids flooding propagation and reduces overhead of establishing routes.

**Figure 1:**
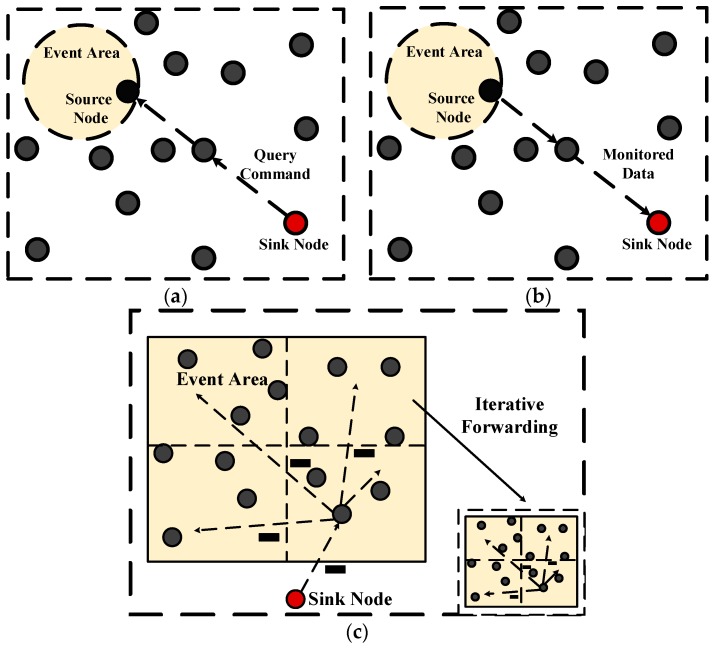
A schematic of Geographic and Energy Aware Routing (GEAR) protocol. (**a**) Process of sending a query command; (**b**) The required data is transmitted to the sink node along the reverse path; (**c**) The query iteration in event area.

Generally, the GEAR protocol utilizes the idea of greedy algorithm in GPSR. The path from the sink node to the event area is built based on the location and energy information of nodes. The spread of packets in GEAR protocol includes three stages: First, the sink node issues a query command. According to the geographical location of the event area, the query command is transmitted to the node which is the closest one to the sink node inside the event area. Then, an iterative forwarding mechanism is triggered to propagate the query command from the node to all other nodes in the area. Finally, the monitored data is transmitted to the sink node along the reverse path, which are shown as (a), (b) and (c) in Figure 1.

However, since the GEAR protocol relies on the location information of nodes, the attackers can launch the Sybil attack by forging the location information. Moreover, the GEAR protocol utilizes the remaining energy of nodes as a factor to select the path. The attacker can deceive the surrounding nodes to elect it as the next hop by broadcasting forged energy information. By obtaining the forwarded packets of the surrounding nodes, the attacker can implement the selective forwarding attacks. Inspired by the above issues, we introduce the IN mechanism to the GEAR protocol.

#### 3.1.2. IN Mechanism

**Figure 2:**
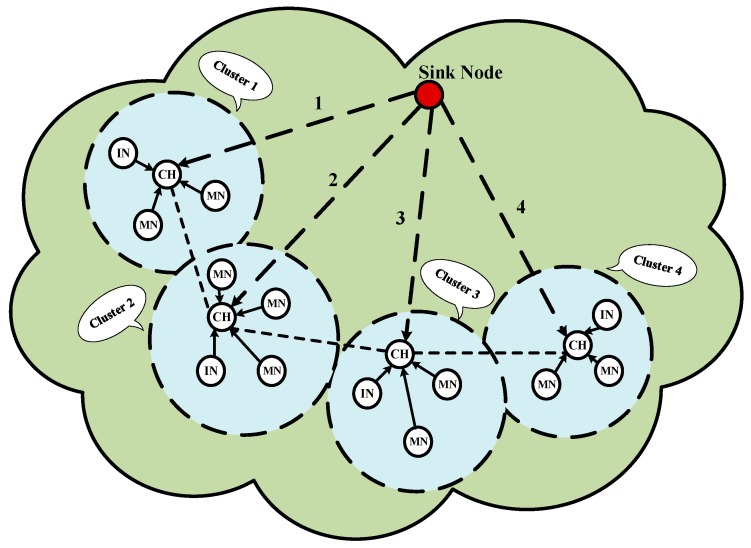
The basic topology of IN mechanism.

The IN mechanism makes use of the packet monitoring strategy to prevent deliberate destruction and malicious attacks. Three types of nodes are involved in the mechanism, i.e., CH nodes, INs and MNs. As shown in Figure 2, they can be supervised and checked by each other. The INs can overhear the CH’s behaviors. Once the IN detects some unusual actions of CHs, it will blacklist the CH and inform the surrounding MNs to stop sending data to the CH. The MNs may refuse the decision, if the decision of the IN is a deliberate accusation. Moreover, the CHs can send random checking requests to INs for adjusting their status. If the MNs have not participated in the IN nomination for long, they will receive a bad reputation. MNs can check if the CH is normally transmitting data and if the IN is working correctly.

(a) Inspecting Nodes

On one hand, the INs play an important role in monitoring the activities of the CHs and the MNs. First, if the IN observes that the CH did not forward more than a fixed number of packets, the IN will send an accusation message to MNs and broadcast the bad reputation of the CH. Then, the CH is placed in the blacklist.

**Figure 3:**
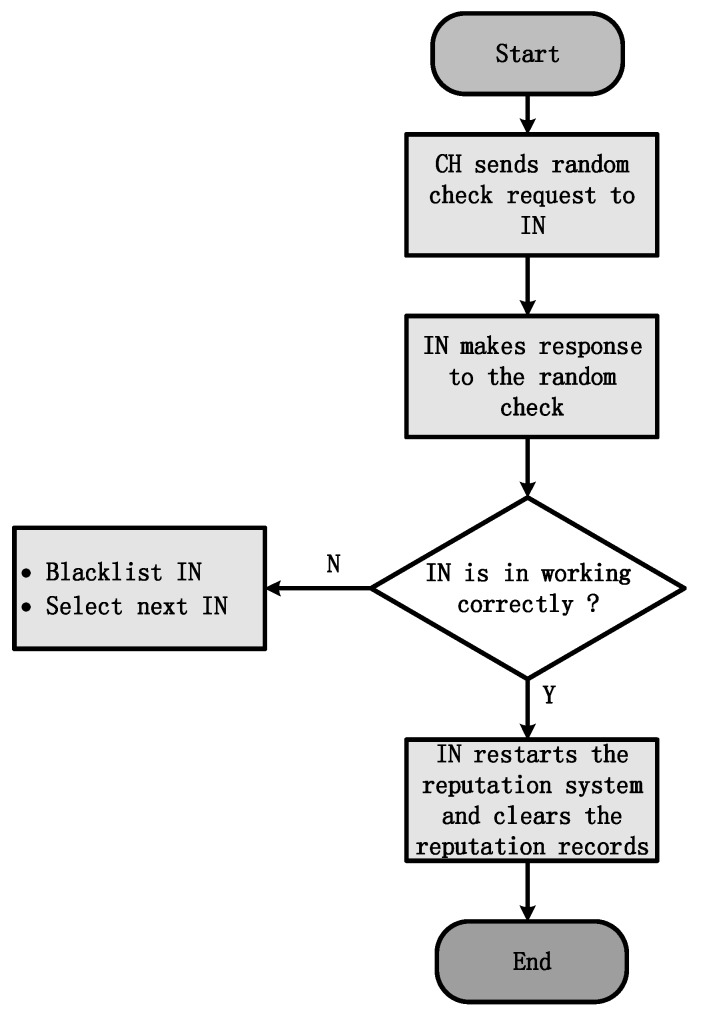
Cluster Head (CH) node checks the Inspecting Node (IN).

On the other hand, the INs could be checked randomly by the CH for judging their status. The CH sends a historical packet that was supervised before sending to the IN. Since the IN does not know which packet will be requested in the history records of the reputation system, it cannot cheat on the random checking process. The IN will then make a response to the request of CH, which is the same as the previous action. According to the response of the INs, the CH can ensure the IN is working well or not. If it is working correctly, the IN will restart the reputation system. Then, the historical reputation records of the IN will be cleared. The IN will continue to monitor the node actions and keep the monitoring history to satisfy the next random checking request from the CH. If not, the IN will be blacklisted by the CH. The specific processes are shown in Figure 3.

(b) Cluster Head Nodes.

At the initial stage of the network, all nodes should actively participate in the selection of CHs or INs. As this process consumes more energy, a node may avoid this obligation, i.e., not to take part in the selection of CHs or INs. However, the CH can supervise the deliberate action. If the node has refused to serve as a CH or IN for a long time, it will be blacklisted in the reputation system.

If a CH node has not received any penalty for a long time, the IN may exist dishonest actions. If the IN has been working honestly, it replies with the correct response to the random checking of the CH. Once the IN cheats on the CH’s random checking process, it will be also blacklisted in the reputation system.

(c) Member Nodes

The MNs can record the good or bad reputation for CHs and INs. For example, if a CH has been transmitting the packets successfully all the time, the MN will give a good reputation for CH. On the contrary, if a large amount of data is incorrectly forwarded, the CH will be blacklisted in the reputation system.

For the IN, receiving a good or bad reputation depends on whether it can correctly judge the behaviors of CHs. If an IN is not strictly monitored, it is likely to become a selfish node. Then, other nodes will not be accused by the IN even though a lot of packets are not delivered correctly.

### 3.2. Model Establishment

**Figure 4:**
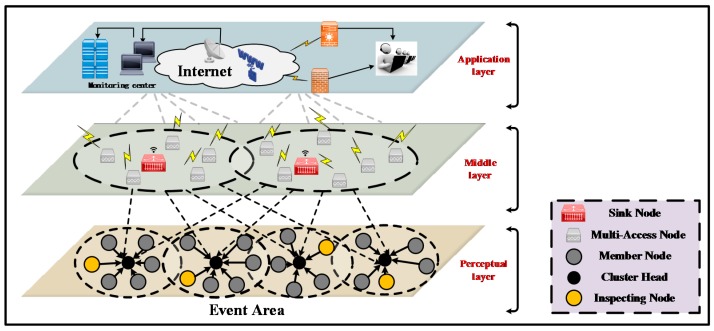
The network model establishment of GIN protocol.

In order to better understand the working mechanism and performance optimization of the GIN protocol, we first establish a network model, and the specific structures of each layer are introduced. As shown in Figure 4, the network model of the GIN protocol is divided into three layers, i.e., application layer, middle layer and perceptual layer.

In the application layer, the managers can learn about the related status of the event regions by collecting data. After the collected information being analyzed, they will take corresponding measures to deal with relevant conditions. Moreover, different monitoring software is added to the application layer according to various service requirements, including energy management, mobile management and assignment management. They are used for monitoring energy usage, nodes movement and task assignment respectively. In terms of the energy management, when the energy of nodes is exhausted, they will broadcast such situation to the surrounding nodes. Upon receiving the information, the surrounding nodes will understand the energy shortage in data forwarding. For the mobile management, the relevant information of nodes can be recorded. For the assignment management, it can coordinate the perceptual tasks of the event area.

The middle layer contains a lot of sink nodes and multi-access nodes. The multi-access nodes are used to receive the information collected from the perceptual layer, and send them to the sink nodes. Meanwhile, these multi-access nodes can also forward the query commands from the upper layer to the bottom layer, while the sink nodes can conduct data fusion and analysis from multi-access nodes, the refined data is sent to the upper layer. Furthermore, the sink nodes will also forward the query commands from the upper layer to the multi-access nodes.

In the perceptual layer, it is the basis of the WSN. It consists of massive scattered sensor nodes. The various sensors can obtain related packets of the event area (such as temperature, air quality, pressure, light, vibration, humidity, etc.) and deliver the collected data to the middle layer. They follow the query command of the upper layer to gather the corresponding information. In the GIN protocol, three kinds of nodes are deployed in the perceptual layer, i.e., MN, CH, and IN nodes. They are supervised by each other and related geographic location messages are exchanged among them.

### 3.3. Primary Processes

In this part, the main implementation processes of GIN protocol are discussed in detail. They contain the clustering behavior, IN election, distributing the reputation system, creating the query command path, spreading the query command in the event area and reverse transmission of collected data. The specific processes are as follows.

**A. Clustering Behavior**

**Figure 5:**
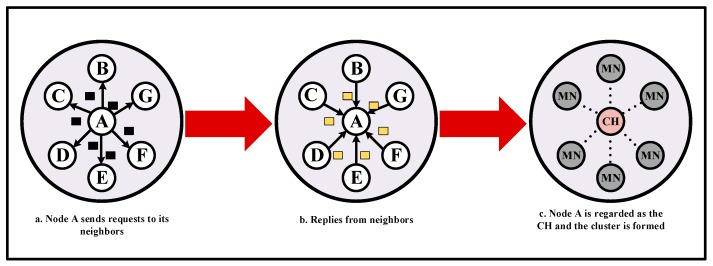
The process of clusters establishment.

The proposed clustering behavior is conducted by two rounds, i.e., the election of CH and cluster formation. A reliable CH selection mechanism can improve the security in WSN [47]. In the first round, if a node wants to be a CH, it will send a request to its one-hop neighbors. Then, the neighbors respond based on previous behaviors of the node. If the number of supporting replies exceed a specific threshold value, the node will be regarded as a CH node. As shown in Figure 5.

In the second round. After the CH node is selected, the remaining nodes will automatically establish the connection with the CH. Finally, the residual nodes serve as the MNs of the CH nodes and the cluster is formed.

**B. IN Election**

In this part, the CH node stipulate that only its MNs can start the IN campaign. Because the IN node must be within the communication range of CH to monitor its behaviors. The selection of the CH and IN is crucial to improve the network life-cycle and energy efficiency [48,49]. In the GIN protocol, the IN election is based on a non-cooperative and repeated game theory. The game is expressed as:

(1) G={N,S,U}

The players are represented by *N*; each player has the same strategy space named *S*; the utility of them is given by *U*. The selection of the strategies is to become an IN or a MN, and the set of strategies can be represented as:

(2) S={IN,MN}

The process of the game can be described as a cost and payment model. If a node chooses one of the strategies, it will get a specific payoff. All the nodes look forward to selfishly select the strategy for maximizing their payoff and consuming the least energy. If a node decides to work as an IN, it can obtain more responsibilities. On the contrary, refusing to be an IN can save its power. Therefore, the set of utility functions is described as:

(3) $$U\left(s_{i}\right)=\left\{ \begin{array}{ll} 0 & \mathrm{when}\;s_{i}=MN,\;\forall_{i}\in N\\ \frac{1}{C_{in}} & \mathrm{when}\;s_{i}=IN\\ \frac{1}{C_{mn}} & \mathrm{when}\;s_{i}=MN \end{array}\right.$$

where *C_in_* and *C_mn_* represent the cost of being the IN and MN, respectively. Then, we can obtain *C_in_* < *C_mn_*. For example, there are two players joining in the game. The payoffs of them are described in Table 1. According to the payoff matrix, if the first player selects to be the strategy IN, the other player will only choose the strategy MN, because the strategy combination (IN,MN) receives more payoffs for the second player, i.e.,

(4) $$\frac{1}{C_{mn}}>\frac{1}{C_{in}}$$

If not, the other player will select the strategy IN, because the strategy pairs (MN,IN) receives more payoffs for the second player than (0,0), i.e.,

(5) $$\frac{1}{C_{in}}>0$$

In another case, if the selection order is changed, both players will still choose the strategy pairs (IN,MN) and (MN,IN). The payoffs of the selection will be

(6) $$\left(\frac{1}{C_{in}},\frac{1}{C_{mn}}\right)$$

and

(7) $$\left(\frac{1}{C_{mn}},\frac{1}{C_{in}}\right)$$

Therefore, the best result is that one player decides to be an IN and another selects to be a MN, which is a pure Nash Equilibrium.

**Figure 6:**
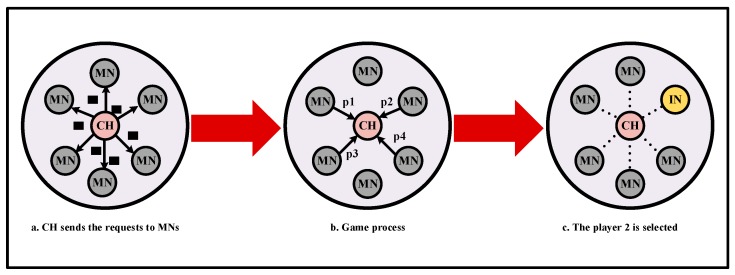
The process of the IN selection.

The game schematic is shown in Figure 6.

**C. Distributing the Reputation System**

The inside attacks from authorized nodes are more difficult to be detected comparing to the outside ones from unauthorized nodes. Therefore, the reputation system is used for solving this issue by giving penalties to the malicious nodes. In other words, the attackers will be blacklisted in the reputation system.

In the GIN protocol, each node is equipped with a reputation function to supervise the behaviors of surrounding nodes. According to the actions of forwarding data, clustering process and monitoring nodes, the system can record reputation values of neighboring nodes. The system can reserve them to give a reference for new nodes. The specific reputation management mechanism is shown in Table 2. The IN is responsible for the behavior supervision of CH and MN. The CH is in charge of the reputation of IN, MN and neighbor CHs. The MN can give an evaluation to the IN and CH. The supervision behavior takes place during the entire process of sending and receiving packets.

**D. Creating the Query Command Path**

In the GIN protocol, when the node *N* needs to transmit the query command *P* to the event area *R*, the next hop of the node *N* is selected based on the shortest distance from the neighboring nodes to *R* and the energy consumption of the neighboring nodes is also taken into account. In order to comprehensively consider the distance and energy, the proposed protocol utilizes the learned cost and estimate cost to identify the path cost. The *h*(*M,R*) is described as the learned cost from the node *M* to node *R*. When the node does not know the learned cost, *c*(*N_i_,R*) substitutes the learned cost for representing the estimate cost of the neighboring nodes. We can get:

(8) $$c\left(N_{i},R\right)=ad\left(N_{i},R\right)+\left(1-a\right)e\left(N_{i}\right)$$

where *ɑ* is a variable parameter, *d*(*N_i_,R*) is regarded as the distance from *N_i_* to the center *D* of the event area *R*, and e(*N_i_*) is named as the energy consumption of *N_i_*. The distance *d*(*N_i_,R*) and consumption e(*N_i_*) are all the normalized formulas. When the node *N_i_* is selected as the next hop of *N*, the learned cost of *N* becomes:

(9) $$h\left(N,R\right)=h\left(N_{i},R\right)+c\left(N,N_{i}\right)$$

where *c*(*N,N_i_*) is described as the transmission consumption from *N* to *N_i_*.

**Figure 7:**
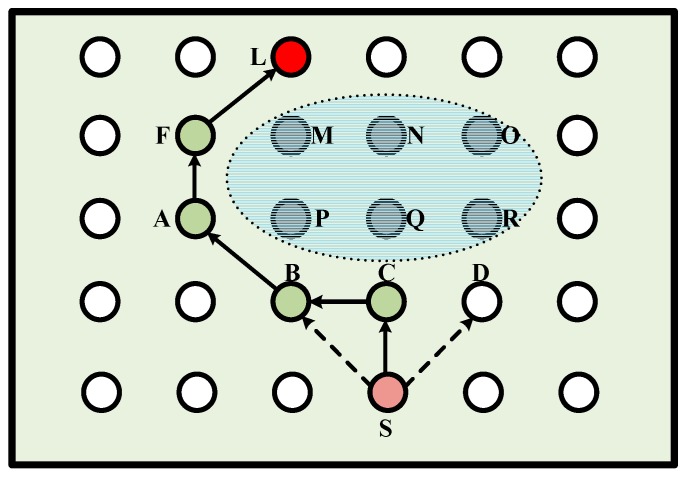
The hole problem of routing path.

When there exists a hole problem in the routing process, as shown in Figure 7, we assume that the distance between the adjacent nodes is *i* and each node can communicate with the eight surrounding nodes. The six nodes in the figure (i.e., *M*, *N*, *O*, *P*, *Q*, *R* nodes) cannot forward the packets because of being used up or attacked. The packets are to be transmitted to the destination node *L*. The GIN protocol is based on the greedy algorithm of the GPSR. When the node *S* receives the query command, the node *S* selects node *C* among three nodes *B*, *C*, *D* as its lowest-cost neighbor. Because node *C* is the closest point to *S* on the transmission path from node *S* to node *L*. In order to solve the routing hole problem, node *C* chooses the neighboring node with the lowest learned cost as the next hop node. In this situation of Figure 7, node *B* is selected as the next hop of node *C*. The learned cost from node *C* to the destination is changed to *h*(*C,L*) = *h*(*B,L*) + *c*(*C,B*). According to the routing rule, a secure query command path will be established.

**E. Spreading the Query Command in the Event Area**

After the query command is transmitted to the event area, the GIN protocol utilizes the iterative geographic forwarding strategy to spread the command in the event area. The node that first received the query command divides the area into multiple sub-areas and forwards the query command to the central location of all sub-areas. In each sub-area, the node closest to the center of the area can receive the query command. Then it will subdivide the sub-areas into more sub-areas and forward the command. When a sub-area has no nodes, the entire iteration process is finished and query command is diffused to the total event area.

**F. Reverse Transmission of the Collected Data**

**Figure 8:**
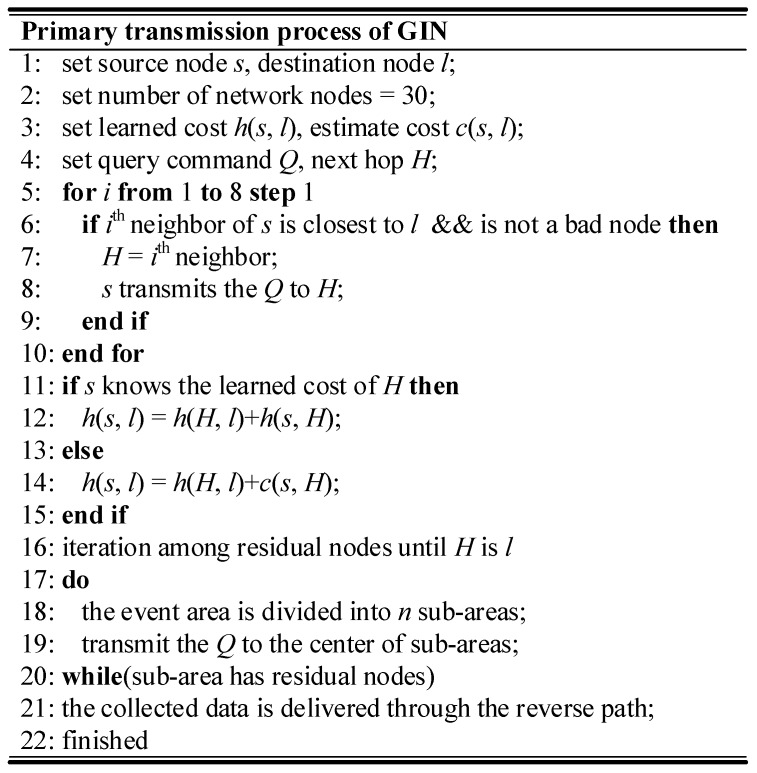
The transmission process in GIN.

After the query command is transmitted to the total event area, the nodes will transmit the monitoring data in the opposite direction of the query path. The learned cost of each-hop node is attached to the monitoring data. For each node, the energy cost is first recorded in the incidental information. Then, this energy cost is added to the consumption value from the current node to the next-hop node. The calculation result will replace the original consumption value. When the node forwards the query command next time, it will replace the formula *d*(*N,R*)with updated learned cost. The nodes can select an optimal path to transmit the data to the event area. When the monitoring data is sent back to the sink node, the transmission process is over. The pseudocode of the transmission process is illustrated in Figure 8.

## 4. Validation and Discussion

In this section, the network topology is established in the simulation environment. Based on a large number of discussions and analyses, the proposed protocol is evaluated and verified. We first set the scenes and parameters of the simulation system, then the experimental results are illustrated based on delay, packet loss ratio and throughput respectively.

**(a) Simulation Setup**

To better evaluate the performance of the proposed protocol, we use the NS3 simulator and Wireshark tools to provide experimental results. The simulation environment is divided into five scenarios, i.e., consisting of 40, 80, 120, 160, 200 nodes in the range of 200 m × 200 m with four clusters in the network. We design the topology of the WSN and set the intrusion scenarios of attack nodes. In this case, we take one cluster of them as an example. The nodes are deployed randomly in the network and multiple compromised nodes (such as the black hole, wormhole and Sybil attacks) are set in the network topology. According to the working principle of different protocols, they have diverse ways dealing with attacking events. Therefore, the routing performances of them are different. For the flooding protocol, when some nodes are attacked in the network, the flooding mechanism is to spread a packet to its surrounding nodes and perform this operation repeatedly until the target area receives the packet. This “best effort” approach is inefficient and energy-consuming. Moreover, the most important thing is that this method cannot sense the existence of attacks. For the two geographic routing protocols (GEAR and GPSR), although they can discover the compromised nodes, the routing performances are much worse than that of GIN protocol. In the simulation, the GIN protocol can detect the attacks as early as possible and dynamically select the next hop, which effectively bypasses the attack area and improves the network performances. The specific parameters are sent as shown in Table 3.

**(b) Analysis of Simulation Results**

Based on the above contents, the simulation results of the three parameters (i.e., delay, loss packet ratio and throughput) are discussed respectively.

**Figure 9:**
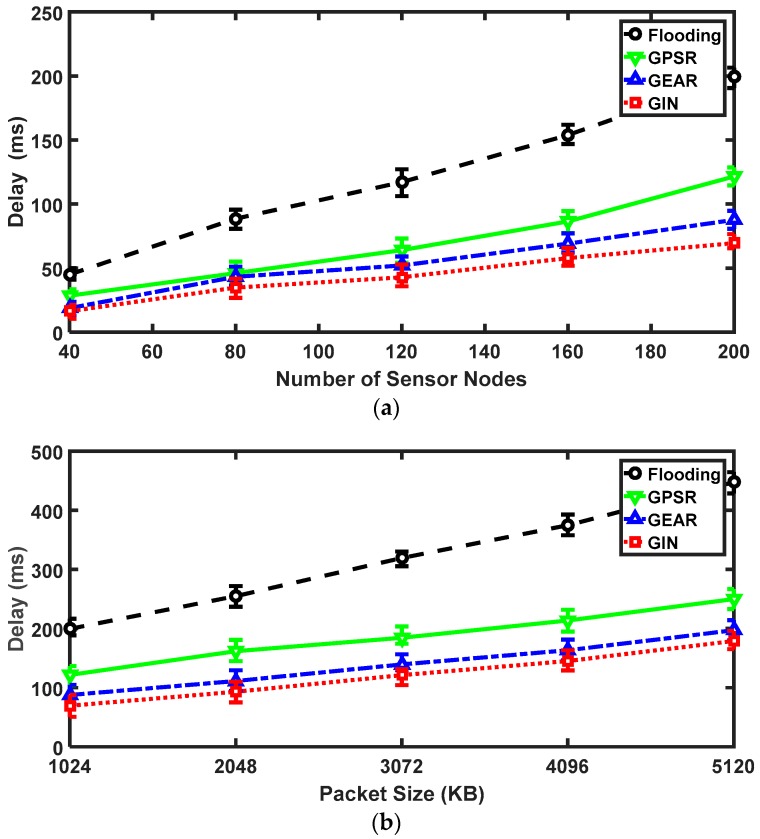
The results for GIN in delay. (**a**) Relationships between the delay and the number of nodes; (**b**) Relationships between the delay and the packet size.

For the delay aspect, it is related to the number of sensor nodes and the packet size. As shown in Figure 9a. With continuous expansion of the network scale, the delay of all protocols gradually increases. Due to a large number of data forwarding in the flooding method, the delay problem is particularly serious. Its delay is higher than that of other protocols. From the 40 nodes to 200 nodes, the delay in flooding protocol has increased proportionately. When there are 200 nodes in the network, the delay is even higher than 200 ms. This result has far exceeded that of the other three protocols. While, the latency of the other three protocols is almost identical between the 40 nodes and 80 nodes. The delay performances of them are all below 50 ms. Starting from 120 nodes, the gap of the three protocols has gradually emerged. The growth rate of GPSR starts to accelerate. In short, the GIN protocol outperforms the other three protocols in the delay aspect. It utilizes the least time to obtain monitoring information and maintains a stable growth of delay. In Figure 9b, the delay is affected by the size of packet. The packet size is divided into five scenarios (i.e., 1024 KB, 2048 KB, 3072 KB, 4096 KB and 5120 KB). We can see that the flooding still exists in the largest delay, which is much larger than that of others. Starting from 1024 KB, the delay of flooding exceeds 200 ms and continues to grow. When the packet size approaches to maximum, the delay of that is more than 400 ms. While, the delay of other three protocols is only around 200 ms at the maximum packet size. Moreover, the delay levels of GEAR and GIN protocols are very similar. The difference is that the GIN is equipped with the IN mechanism. Among the four protocols, the GIN protocol consumes the least time to complete data collection, which can improve transmission efficiency and reduce the number of retransmissions.

**Figure 10:**
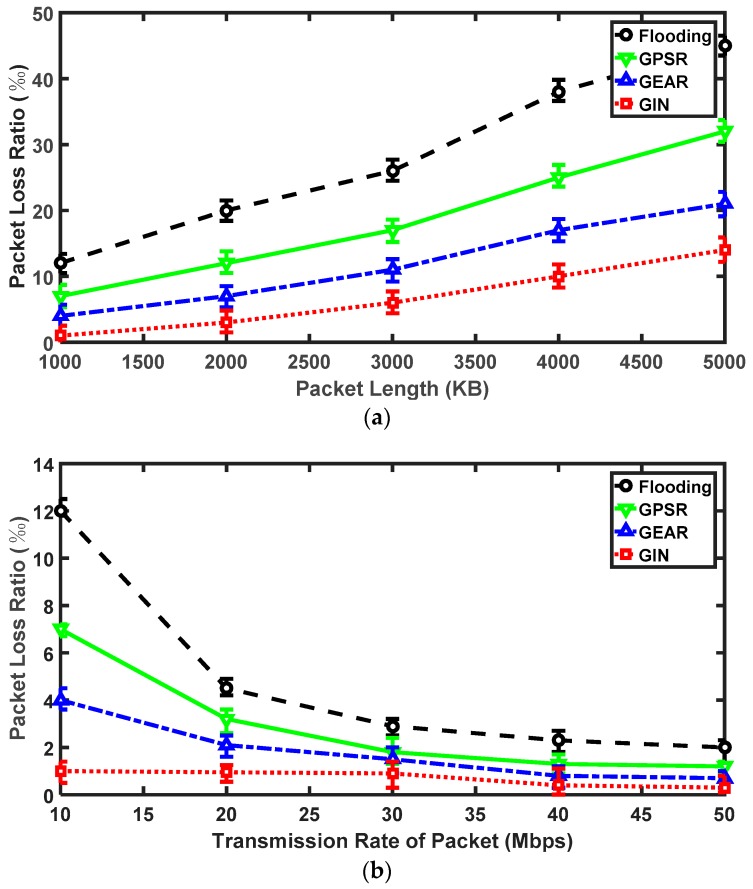
The results for GIN in packet loss ratio. (**a**) Relationships between the packet loss ratio and the packet length; (**b**) Relationships between the packet loss ratio and the transmission rate of packet.

For the packet loss ratio, it is related to the packet length and the transmission frequency. In terms of the packet length, there are five cases (i.e., the packet length is divided into 1000 KB, 2000 KB, 3000 KB, 4000 KB and 5000 KB). As the packet length increases, the packet loss ratio continues to deteriorate as shown in Figure 10a. Especially in the case of the flooding, the packet loss ratio increases steadily between 1000 KB and 3000 KB. However, this number is seriously higher than other protocols. After the 3000 KB line, the packet loss ratio of flooding protocol becomes worse. When the packet length is 5000 KB, the packet loss ratio approaches 0.05. At this stage, the performance of GIN in packet loss ratio is still a preferred one. The maximum packet loss ratio in GIN protocol is almost equal to that of the minimum value in flooding method, i.e., it is approximately 0.01. Moreover, the value gap of GIN and GEAR protocols is almost similar to the gap of GEAR and GPSR protocols in the packet loss ratio. The gap of them is about 0.008. Due to the IN mechanism, the GIN protocol rarely encounters the selective dropping packet attacks. Therefore, it is the safest one of the four protocols. Figure 10b illustrates that the packet loss ratio is inversely proportional to the transmission frequency of packet. When the transmission frequency of packet is 10 Mbps, the packet loss ratio of the four protocols is at their respective maximum. Between 10 Mbps and 20 Mbps, the packet loss ratio of flooding, GPSR and GEAR protocols drops significantly. After 20 Mbps, the downward trend of them tends to be smooth. Conversely, the GIN protocol begins to decline slowly and speeds up later. When the transmission frequency of packet exceeds 40 Mbps, the packet loss ratio of the GIN, GEAR and GPSR protocols is almost the same. However, the value of flooding is still higher than other protocols. In one word, the GIN protocol outperforms other three protocols in the packet loss ratio.

**Figure 11:**
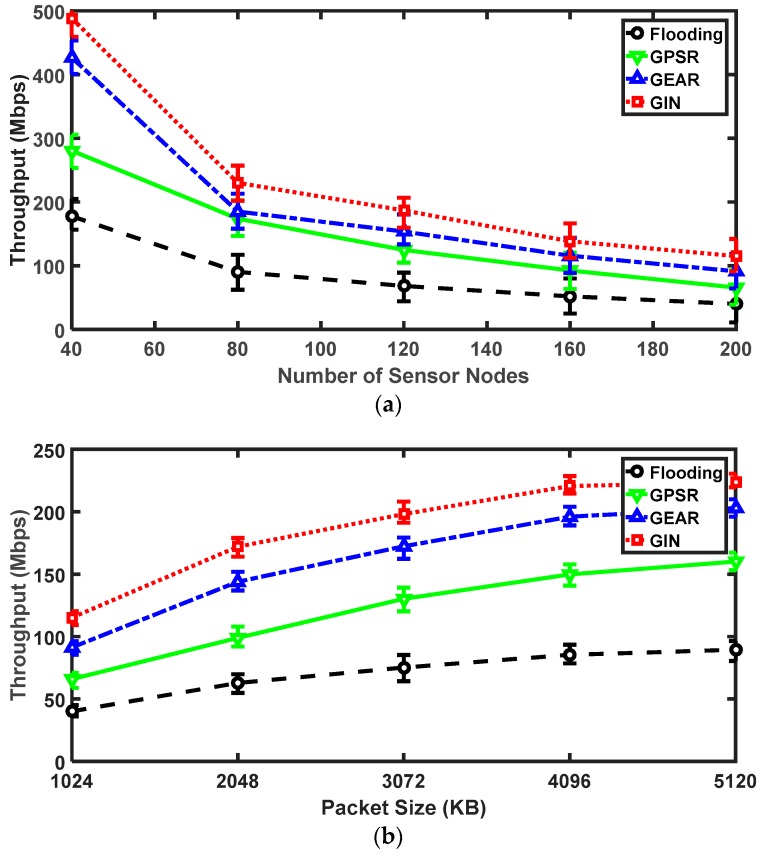
The results for GIN in throughput. (**a**) Relationships between the throughput and the number of sensor nodes; (**b**) Relationships between the throughput and the packet size.

As to the throughput aspect, the number of sensor nodes and packet sizes are two significant factors. In Figure 11a, as the number of sensors continue to increase, the throughput of the four protocols gradually declines. For the GIN and GEAR protocols, they have similar trends in throughput, while the GPSR and flooding protocols exist the analogous downward trend. Before the number of nodes hitting 80, the values of the four protocols all fall quickly. However, as the number of nodes exceeding 80, the speeds of decline all becomes smoother. From an overall perspective, GIN protocol outperforms the other three. Particularly in the scenario of 200 nodes co-existing, the value of GIN protocol is still around 120 Mbps, which is about three times that of the flooding protocol and two times that of the GPSR protocol. From Figure 11b, we can arrive at the conclusion that the throughput is proportional to the packet size. The upward trends of the GIN and the GEAR protocols are basically similar. However, the value of the GIN protocol is higher than that of the other three protocols. The gap between the GPSR and flooding protocols gradually increase starting from 1024 KB, where the flooding protocol has the worst performance. The maximum of the flooding protocol is less than the minimum value of the GIN protocol. When the packet size reaches 5120 KB, the throughput of the GIN protocol becomes three times higher than that of the flooding protocol. Due to the IN mechanism, plenty of packets are securely delivered to the event area.

## 5. Conclusions

For the complex network environments, the IoT is facing serious challenges in terms of security. In this paper, we first discussed the existing wireless routing protocols (i.e., based on geographic location, hierarchy, and data) in detail. To cope with the black hole and Sybil attacks, we proposed a novel GIN protocol, which combined the GEAR protocol and the IN mechanism. Each node in the network was equipped with a reputation function that contained behavioral records of all nodes (i.e., IN, MN and CH nodes). When a node was found to forward packets selectively, the transmission of the information will be terminated. Moreover, the information of energy and location can be exchanged by two neighboring nodes. The GIN protocol utilized the information for selecting the optimal path to the event area. Once the location of a neighboring node was found far beyond the normal signal transmission range, it will be blacklisted and given a bad reputation. The specific model establishment and transmission processes were addressed. Moreover, we utilized the game theory to select INs. To further validate the performances of GIN protocol, we established a network simulation environment by NS3. The results illustrated that our scheme outperformed the other protocols in the network delay, packet loss ratio, and throughput. Comparing with the traditional routing protocols (i.e., flooding, GPSR, and GEAR): the network delay can be further reduced by 67.7%, 43%, and 20%, respectively. The packet loss ratio can be roughly decreased by 68.9%, 56.3%, and 33.3%, respectively. The throughput can be improved by 65.2%, 43.5%, and 20.9% respectively.

The malicious attack issue had seriously obstructed the development of IoT. A smart, collaborative, and reliable routing protocol could make significant changes for information collection procedures. In the future, we will work on the application of the proposed protocol in multiple scenarios.

## Figures and Tables

**Table 1:** The strategy form of two players.

	IN	MN
IN	$\frac{1}{C_{in}}$,$\frac{1}{C_{in}}$	$\frac{1}{C_{in}}$,$\frac{1}{C_{mn}}$
MN	$\frac{1}{C_{mn}}$,$\frac{1}{C_{in}}$	0,0

**Table 2:** The reputation management mechanism.

Type of Node	List of Managed Entities
IN	CH, MN
CH	IN, MN, neighbor CHs
MN	IN, CH

**Table 3:** Simulation parameters.

Parameter	Value
Number of Sensor Nodes	40/80/120/160/200
Network Size	200 m × 200 m
Packet Size	1 M/2 M/3 M/4 M/5 M
Inspection Nodes	4
Cluster Head	4
Number of Packets	1000
Frequency of sending packets	10 Mbps/20 Mbps/30 Mbps/40 Mbps/50 Mbps
